# Architecture and synthesis of P*,*N-heterocyclic phosphine ligands

**DOI:** 10.3762/bjoc.16.35

**Published:** 2020-03-12

**Authors:** Wisdom A Munzeiwa, Bernard Omondi, Vincent O Nyamori

**Affiliations:** 1 School of Chemistry and Physics, University of KwaZulu-Natal, Westville Campus, Private Bag X54001, Durban 4000, South Africa; 2 School of Chemistry and Physics, University of KwaZulu-Natal, Pietermaritzburg Campus, Private Bag X01, Scottsville, Pietermaritzburg 3201, South Africa

**Keywords:** ligand synthesis, P,N-heterocyclic phosphines

## Abstract

Diverse P,N-phosphine ligands reported to date have performed exceptionally well as auxiliary ligands in organometallic catalysis. Phosphines bearing 2-pyridyl moieties prominently feature in literature as compared to phosphines with five-membered N-heterocycles. This discussion seeks to paint a broad picture and consolidate different synthetic protocols and techniques for N-heterocyclic phosphine motifs. The introduction provides an account of P,N-phosphine ligands, and their structural and coordination benefits from combining heteroatoms with different basicity in one ligand. The body discusses the synthetic protocols which focus on P–C, P–N-bond formation, substrate and nucleophile types and different N-heterocycle construction strategies. Selected references are given in relation to the applications of the ligands.

## Introduction

Phosphines constitute a large percentage of ligands in organometallic chemistry and over the years, they have received enormous attention. The main interest towards this class of compounds is attributed to aspects such as, the good electron-donating ability of the phosphorous atom, and the ease of optimizing steric and electronic properties. Additionally, properties like chirality can be conferred to the backbone of the ligands to generate C-stereogenic [1] and P-chirogenic [2] compounds. Furthermore, the ^31^P-nucleus abundance allows the use of NMR for reaction monitoring and in situ speciation. In addition, phosphine ligands have found various applications as auxiliary ligands in organometallic transition-metal complexes. A great number have exhibited potential application in organic light-emitting devices (OLEDs) [3], medicine [4–6] and catalysis [1,7–8] among other fields (Table 1). There is a number of review articles in the literature [9–11] which explore deeper into the applications of P,N-heterocyclic phosphine ligands. Besides, the inclusion of other heteroatoms in the phosphine ligand skeleton opens up many possibilities for metal coordination [12]. Thus, their use in catalysis is the basis of this review article with the main focus on the synthesis of N-heterocyclic phosphines.

**Table 1:** Selected works on the applications of P,N-phosphine ligands.

Type of ligand	Application	References

pyridyl phosphines	OLEDs	[13]
	Heck coupling	[14]
	metal organic frameworks	[15]
	polymerization of lactides	[16]
	alkene hydroxylation	[17]
	addition reaction	[18]
	ethylene oligomerization	[19]
	synthesis of pyrazolines	[20]

triazolyl phosphines	Suzuki cross coupling	[21]
	asymmetric hydrogenation	[22]
	luminescence	[23]
	hydroformylation	[24]

pyrazolyl phosphines	coordination polymers	[25]
	Heck coupling	[26]

imidazolyl phosphines	Suzuki coupling	[27]
	hydroamination	[28]
	OLEDs	[29]
	ethylene oligomerization	[30]
	amination	[31]
	olefin metathesis	[32]
	hydroformylation	[33]

pyrrolyl phosphines	hydroformylation	[34–35]
	ethylene polymerization	[36]

oxazolyl phosphines	asymmetric cycloaddition	[37]
	asymmetric hydrogenation	[38]
	carbonylation of alkynes	[39]
	allylic substitution	[40]
	asymmetric addition	[41]
	allylic amination	[42–43]

The presence of soft donor atoms such as phosphorus results in the formation of hemilabile ligands. These are multidentate ligands having hard P-donor and soft N- and/or O-donor atoms [44]. During catalysis the weakly coordinating hard donor atom detaches to give way for the incoming substrate to coordinate to the metal center [45]. This behavior also aids in ligands being able to stabilize low-valent metal states and promote oxidative addition reactions [45–46]. The complimentary effect of P and N can help stabilizing different catalytic species that are produced during catalytic transformations [11,47].

P,N-phosphine ligands can effect regioselective control, due to the *trans*-effect as exhibited in π-allyl metal complexes, where substitution occurs selectively on the end opposite to the phosphorus donor atom [48]. This is because the position *trans* to the heteroatom, with greater π-acceptor character, is more electrophilic than the one opposite the σ-donor atom [9]. One can modify this electronic imbalance by attaching vicinal heteroatoms. The π-acceptor character of phosphorus can be reinforced by the presence of oxygen and/or nitrogen whilst σ-donating potency of nitrogen can be manipulated by switching between sp^3^ and sp^2^ hybridization [9,49–50].

The synthesis of phosphines is quite delicate because when exposed to air, some of them are easily oxidized, hence the reactions are often conducted under inert conditions. Alternatively, the phosphine can be protected as a borane adduct and thereafter, the protecting group is ultimately removed to liberate the free ligand. This method has been developed by Imamoto et al. [51–52] were the phosphine boranes were prepared by reacting phosphines with sodium borohydride. Alternatively, the reduction of phosphine oxide byproducts with lithium tetrahydridoaluminate, calcium aluminum hydride_,_ and hydrosilanes can also be used to regenerate the phosphine ligands. Hydrosilane reagents usually lead to stereoselective reduction products, hence, they are used for the synthesis of chiral phosphines from chiral oxides [53]. Lithium tetrahydridoaluminate is used for the reduction of achiral phosphines because its action on optically active phosphine oxides leads mainly to the optically inactive phosphines ascribed to *pseudo* rotation of the pentacoordinated transition intermediates [52]. Despite this, researchers have synthesized many efficient phosphine ligands, though fast and easy synthetic methods which are principal in the development of flexible ligands are still needed.

## Review

### Preparation of N-heterocyclic phosphines via P–C bond formation

#### Nucleophilic substitution of halogens

There are different methods that have been reported for the construction of the P–C bonds. Two approaches are possible using halogenated precursors. The first one is the organometal-halogen-phosphine route where the metalated organohalogen compound is reacted with the halogen phosphine. Alternatively, the metal phosphide can be reacted with an organohalogen compound leading to the desired product. The most commonly used trans-metalation reagents are Grignard [54] or organolithium reagents [55] and other suitable bases. The metalation reaction is prone to side reactions when carried out at higher temperatures and as such, the reaction must be carried out below 0 °C. For example, pyridyllithium derivatives as intermediates can be subjected to deprotonation, substrate addition and pyridine formation due to lithium halogen elimination, halide migration, and ring-opening reactions [56–57]. Butylphosphines are also formed alongside the main product, and in most cases pure phosphine pyridines are obtained using column chromatography followed by extractions adding to the number of synthesis steps.

**Scheme 1:**
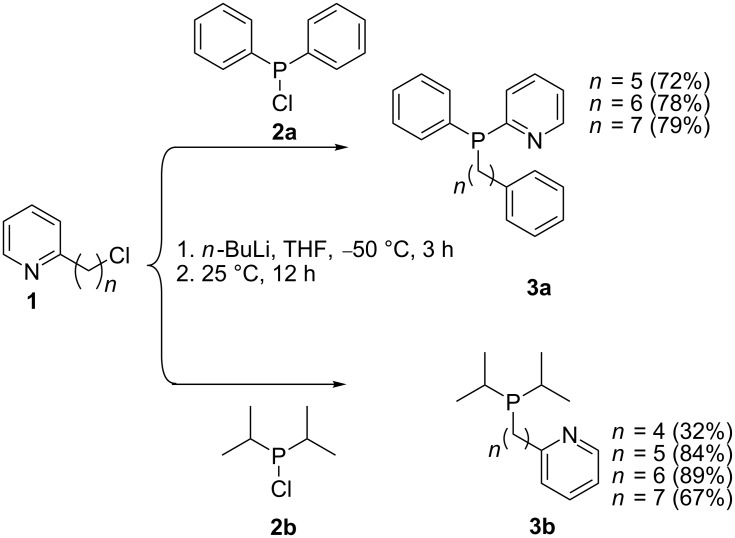
Synthesis of pyridylphosphine ligands.

**Figure 1:**
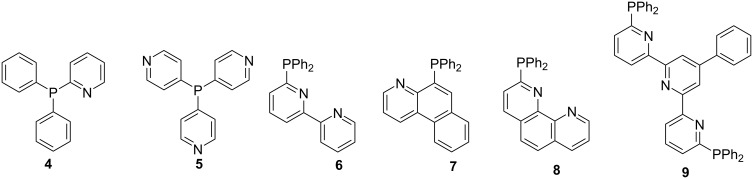
Pyridylphosphine ligands.

This method has proven handy in the synthesis of phosphine pyridyl-type ligands. Jasen et al. [58] reported on the synthesis of picoline analogs by reacting the organohalide **1** with a lithium phosphide generated from chlorodiphenylphosphine (**2**) (Scheme 1). The resulting phosphine ligands **3** were obtained in relatively good yields. Notably, a low isolated yield was reported when starting from 2-(4-chlorobutyl)pyridine (*n* = 4) and this was attributed to the competing cyclization reaction affording cyclic pyridinium salts. The prominent 2-(diphenylphosphine)pyridine (**4**) has proved to be an interesting building block for the assembly of homo and hetero-organometallic complexes. The 3- and 4-pyridylphosphine derivatives **5** have also been successfully used as templates for assembling supramolecular structures and coordination polymers [54,59]. Halogenated ring-fused pyridine reagents can also be used to generate bipyridyl- (**6**), quinolinyl- (**7**), phenanthrolinyl- (**8**) and terpyridinyl- (**9**) phosphine ligands (Figure 1) [60].

Trofimov et al. [61–62] reported on an alternative reaction pathway using microwave heating for the synthesis of tris(2-pyridyl)phosphine in which white and red phosphorus were used. On treating the red phosphorus with 2-bromopyridine in potassium hydroxide/dimethyl sulfoxide emulsion, pyridylphosphine was obtained in moderate yields. Traces of phosphine oxide were present as evidenced by the observation of two phosphorus peaks in the ^15^P NMR spectrum.

An optimized method via Grignard reagents has been reported by Kluver et al. [54], by which the product was isolated in excellent yield (71%). It was noted that the magnesium ions increase the water partition coefficient of these compounds since they coordinate stronger to the nitrogen atoms as compared to lithium ions. In this case, common extraction with dichloromethane was not applicable, hence solid–liquid extraction with diethylamine was used. Low yields were reported for the 3- and 4-pyridyl analogs due to the difficulty associated with their extraction compared to their 2-pyridyl counterparts [54–55].

**Scheme 2:**
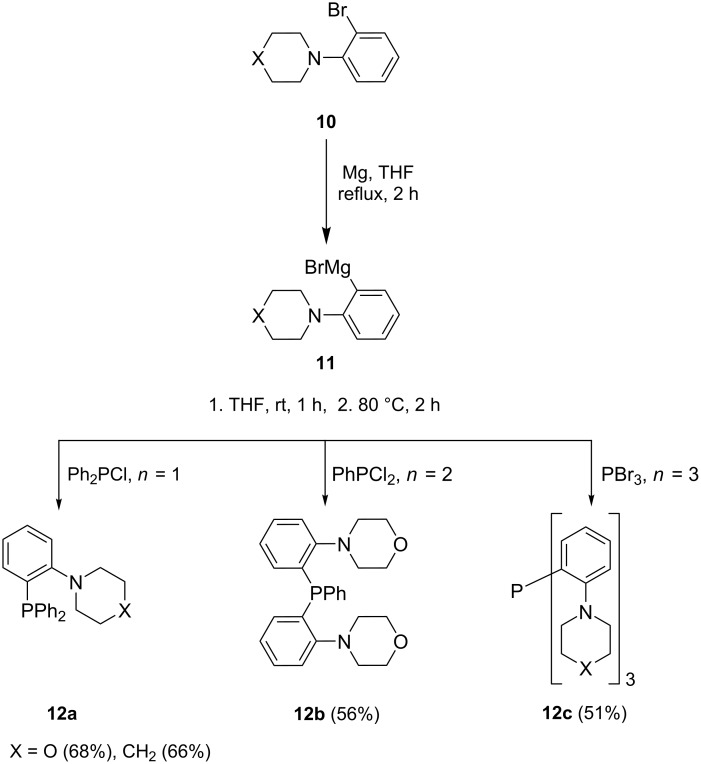
Synthesis of piperidyl- and oxazinylphosphine ligands.

Dai and co-workers [63] also used the Grignard route to synthesize phosphine ligands that are stable to oxidation as described in Scheme 2. The organomagnesium intermediate **11** produced from 2-(*N*-piperidyl)bromobenzene (**10**) was trapped with appropriate halo-phosphine reagents to generate derivatives **12**. The 2-(*N*-piperidyl)phenyl-substituted phosphine (X = CH_2_, *n* = 1) was obtained in relatively good yield while the 2-(*N*-morpholinyl)phenyl derivatives (X = O, *n* = 1, 2, 3) were obtained in moderate yields. The reactions were complete within 3 h despite the fact that the Grignard substrate contains an *ortho*-substituent. This methodology was also faster than the metal-catalyzed phosphorylation route reported by the same authors.

**Scheme 3:**
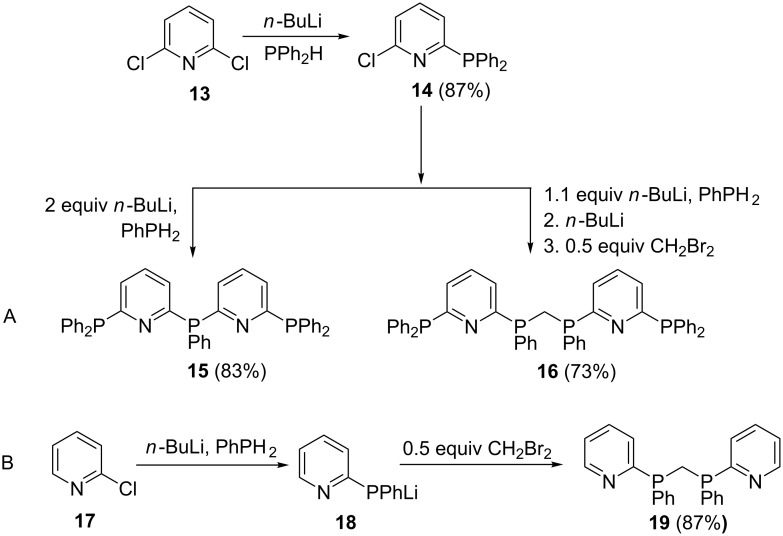
Synthesis of linear multi-chelate pyridylphosphine ligands.

The use of multiply halogenated compounds opens up opportunities to synthesize multidentate ligands. Zhang et al. [64] reported on a sequential synthetic route of multichelate pyridylphosphines **15**, **16** and bis(2-pyridylphenylphosphino)methane (dpypm, **19**) as shown in Scheme 3. Ligands **15** and **16** were prepared from intermediate **14**, which in turn was obtained upon treating 2,6-dichloropyridine (**13**) with the generated lithium phosphide reagent. The phosphine ligand **15** was obtained by reacting chloropyridylphosphine **14** with PhPLi_2_. In a similar manner, the hexadentate pyridylphosphine **16** was synthesized: Firstly, PhPH_2_ was treated with an equivalent amount of *n*-BuLi to afford LiPHPh. The latter was then reacted with **14**, followed by deprotonation with *n*-BuLi and reaction with 0.5 equiv CH_2_Br_2_ to afford hexadentate compound **16** in 73% yield.

The synthesis of **19** (Scheme 3B) was achieved in moderate to high yield, firstly, by reacting equimolar amounts of LiPHPh and 2-chloropyridine (**17**) to give (2-pyridyl)phenylphosphide **18**. Secondly, compound **18** was bridged by reaction with a half molar equivalent of dibromomethane to furnish the desired ligand **19**.

**Scheme 4:**
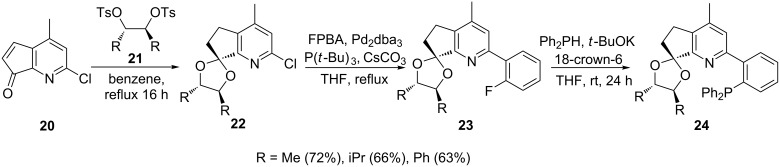
Synthesis of chiral acetal pyridylphosphine ligands.

The nature of the halide in the precursors also influences the reaction progress. Fluorine and chlorine usually require strong bases for the metal–halogen exchange, while relatively mild bases can be used for bromo and iodo derivatives. Structurally inflexible chiral acetal ligands have been reported by Lyle et al. where the fluorine–metal exchange was achieved by treatment with potassium *tert*-butoxide for a relatively long period (24 h) (Scheme 4) [65]. Acid-catalyzed condensation of compound **20** with enantiomerically pure *C*_2_-symmetric 1,2-tosylate analogs **21** (R = Me, iPr and Ph) in benzene produced chiral acetal **22**. Subsequent palladium-catalyzed C–C coupling of the acetal with 4*-*fluorophenylboronic acid (FPBA) in the presence of caesium carbonate and tri-*tert*-butylphosphine afforded aryl fluorides **23**. Pure ligands **24** (63–72%) were obtained by phosphorylation with diphenylphosphine in the presence of potassium *tert*-butoxide and 18-crown-6.

#### The use of silyl and dialkylamine as reagents

**Scheme 5:**
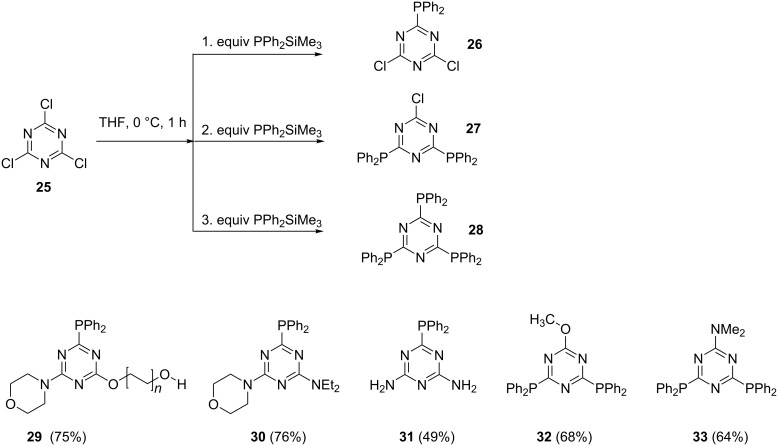
Synthesis of diphenylphosphine-substituted triazine ligands.

Organosilyl, silylphosphine derivatives, along with dialkylamines can also be used as alternative substrates to halogen-based reagents. These compounds are more stable nucleophiles compared to organometallic or metal phosphides generated through metalation processes. Hayashi et al. [66] used tris(trimethylsilyl)phosphine to control the nucleophilic substitution in the preparation of P,N-(phosphino)triazine ligands (Scheme 5). It was shown that the use of other nucleophiles failed to give controlled products, i.e., when lithium phosphide was used in a 1:1 ratio a mixture of products was obtained. A reaction between one molar equivalent of cyanuric chloride (**25**) and tris(trimethylsilyl)phosphines formed the unstable monophosphine intermediate **26**, which was isolated as amino and/or alkoxy derivatives **29–31**. Selectively varying the molar ratio of the silylphosphine nucleophile and the starting reagent **25** resulted in the corresponding bis- and tris(diphenylphosphine)triazine motifs **27** and **28**. A subsequent nucleophilic substitution reaction of **27** gave compounds **32** and **33**.

**Scheme 6:**
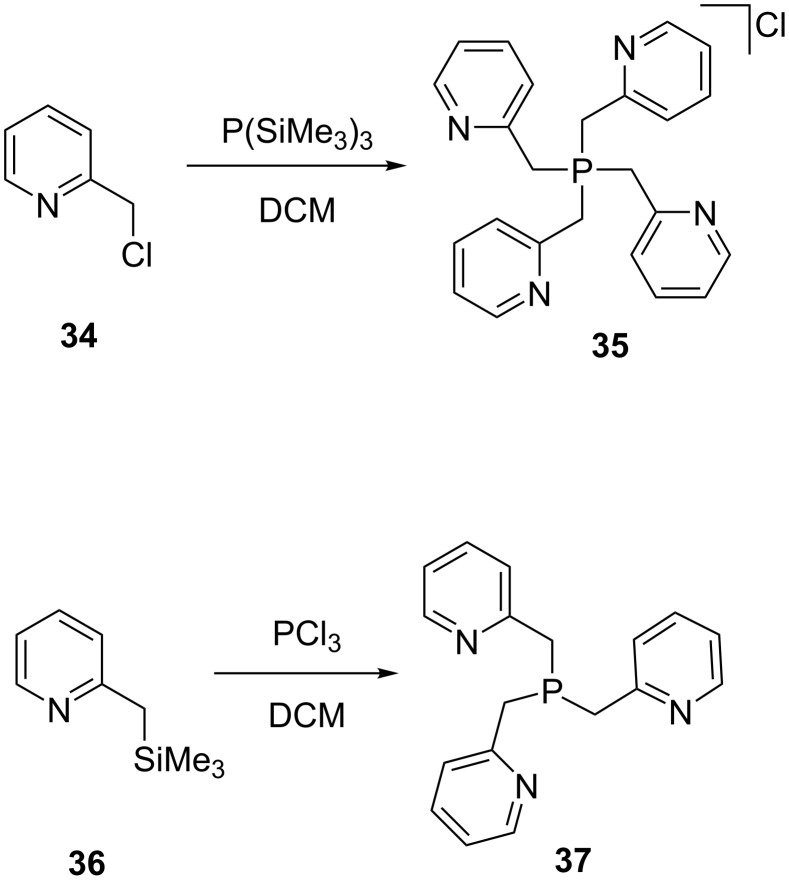
Synthesis of (pyridine-2-ylmethyl)phosphine ligands.

Changing the silylated substrate can also influence the product formed. Thus, when reacting P(SiMe_3_)_3_ with 3 equivalents of 2-picolyl chloride (**34**) in DCM ligand **35** was obtained in low yield (33% based on **34**). Alternatively, when 3 equivalents of 2-(trimethylsilylmethyl)pyridine (**36**) with an equivalent of phosphorous trichloride were reacted, compound **37** was obtained with relatively good yield of about 76% (based on PCl_3_, Scheme 6) [67]. The byproduct Me_3_SiCl can be easily be removed by distillation or in vacuo.

**Scheme 7:**
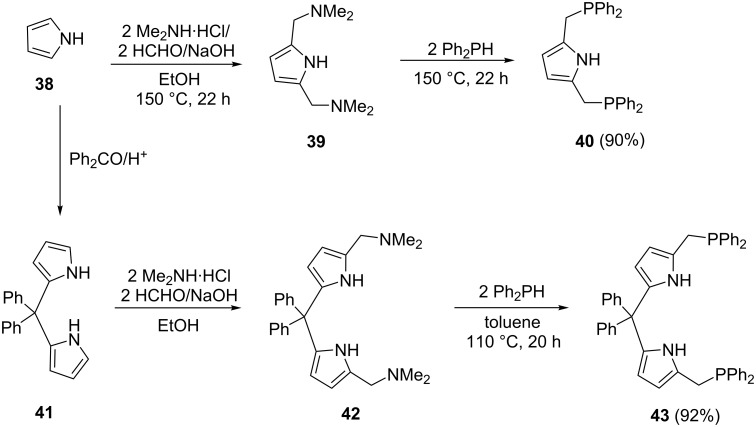
Synthesis of diphosphine pyrrole ligands.

A facile substitution of dimethylamine with phosphine in the synthesis of P,N,P and P,N,N,P pyrrolylphosphine ligands **40** and **43** was reported by Kumar et al. [68] (Scheme 7). The condensation of pyrrole (**38**) with formaldehyde and the amine gave the bis(diaminomethyl)pyrrole **39**, which on reaction with Ph_2_PH gave diphosphine pyrrole ligand **40** in good yield (90%). Following a different route, condensation of pyrrole (**38**) with diphenylketone gave diphenyl(dipyrrolyl)methane **41**. Subsequent Mannich condensation reaction resulted in the pyrrole diamine **42**. Refluxing a toluene solution of intermediate **42** and diphenylphosphine gave dipyrrolyldiphosphine ligand **43** in very good yield (92%). Generally, high temperatures are involved, and the reaction requires relatively longer times compared to the organometallic route.

#### Reaction of metal phosphides with cycloalkanes

**Scheme 8:**
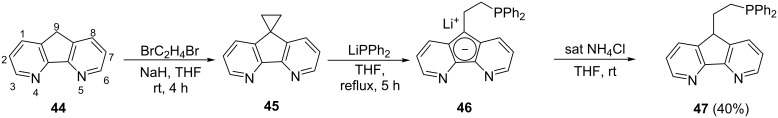
Synthesis of 4,5-diazafluorenylphosphine ligands.

Cyclopropane easily undergoes nucleophilic substitution reactions because of its high ring strain. Tan et al. [69] reported the preparation of 9-(2-(diphenylphosphine)ethyl)-4,5-diazafluorene ligand **47** which includes a cyclopropylated intermediate (**45**, Scheme 8). The ligand was prepared by an initial cyclopropanation of diazafluorene **44**. For this, **44** was treated with a dibromomethane solution in THF in the presence of sodium hydride under reflux for four hours to obtain cyclopropyl intermediate **45**. The latter was converted into compound **46** by reaction with lithium diphenylphosphide in dry THF. Finally, the desired ligand **47** was obtained after quenching the intermediate compound **46** with a saturated solution of ammonium chloride. Ligand **47** was investigated for its ability to undergo ligand transfer reactions.

**Scheme 9:**
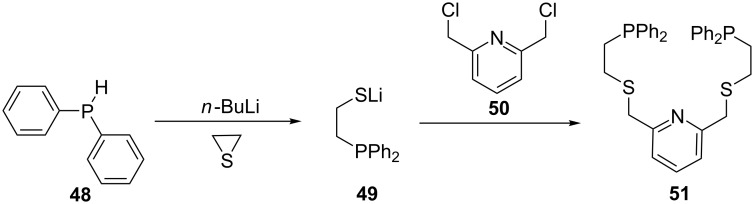
Synthesis of thioether-containing pyridyldiphosphine ligands starting from ethylene sulfide and diphenylphosphine.

Ethylene sulfide has also been used as precursor for the synthesis of phosphine ligands. Kuang et al. synthesized a thioether-functionalized pyridine-based diphosphine ligand starting from diphenylphosphine and ethylene sulfide (Scheme 9) [70]. Thus, the diphosphine ligand **51** was obtained in good yield by reacting 2,6-bis(chloromethyl)pyridine (**50**) with phosphine lithiothiolate **49**. The latter was obtained by treatment of diphenylphosphine (**48**) with *n*-BuLi and ethylene sulfide in tetrahydrofuran at very low temperatures.

#### Metal–proton exchange from α-C–H bond activation in heterocycles

**Scheme 10:**
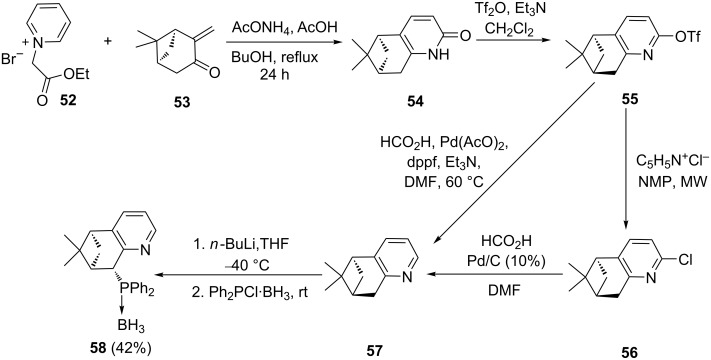
Synthesis of monoterpene-derived phosphine pyridine ligands.

The α-position to a heteroatom in a cyclic compound is activated because of the difference in electronegativity with carbon. This presents an opportunity to readily generate organometallic nucleophiles. Chelucci et al. [71] used this fact to synthesize the monoterpene-derived pyridylphosphine ligand **58** (Scheme 10). The key step was a Kröhnke annulation reaction. The Kröhnke salt **52** was pre-synthesized from ethyl bromoacetate and pyridine and then reacted with (−)-pinocarvone (**53**) in the presence of ammonium acetate. The obtained keto intermediate **54** was then treated with triflic anhydride to afford the corresponding triflate **55**. Microwave-assisted reduction of compound **55** with pyridinium chloride afforded the α-chloropyridine derivative **56**, which was further catalytically dehalogenated with palladium on carbon and formic acid to generate the pyridine scaffold **57**. Coupling of **57** with Ph_2_PCl·BH_3_ resulted in the boron-protected ligand **58**, which was deprotected with Et_3_N. Alternatively, 1,1’-bis(diphenylphosphino)ferrocene (dppf) with palladium(II) acetate was used to catalyze the reduction of **55** generating the pyridine scaffold **57**. Subsequent lithiation and addition of chlorophosphine resulted in the desired ligand **58**. However, the overall yield was lower than the yield obtained through the other method.

**Scheme 11:**
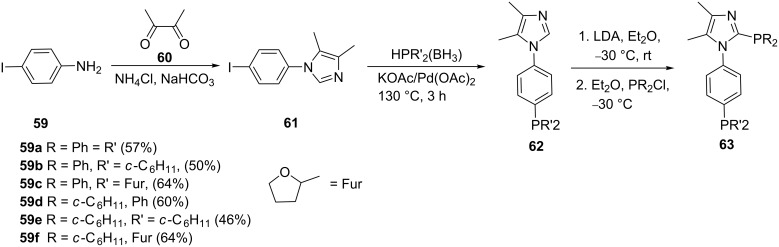
Synthesis of *N*-phenylphosphine-substituted imidazole ligands.

Imidazole can be regioselectively deprotonated at the more acidic C_2_ position. The mono- and diphosphine imidazole ligands **62** and **63** were conveniently synthesized by Milde et al. (Scheme 11) [8]. The imidazole intermediates **61** were obtained by coupling iodoaniline (**59**) with the dialdehyde **60**. Selective metalation of the imidazole ring and subsequent treatment with the phosphine gave the imidazolylphosphine ligands **62** and **63** (46–64%).

**Scheme 12:**
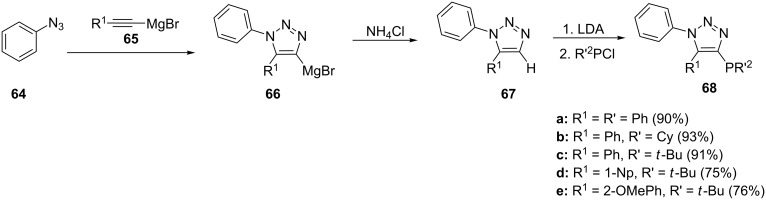
Synthesis of triazol-4-ylphosphine ligands.

The fast and clean alkyne–azide cycloaddition reaction has been applied successfully to prepare click-phosphine ligands [72]. The presence of three nitrogen atoms within the five-membered ring results in a high activation of the α-position and the highly acidic nature of the proton makes it easy for abstraction. Sharpless et al. [73] reported on the synthesis of 1,5-disubstituted triazoles and Liu et al. [74] used this procedure to synthesize triazolylphosphine ligands with the phosphorous substituent in the α-position (Scheme 12). For this, the aryl azide **64** was reacted with bromomagnesium acetylides **65** to generate magnesium-containing triazoles **66** which, upon quenching with ammonium chloride, afforded the triazoles **67**. Lithiation followed by coupling with the appropriate chlorophosphines resulted in the desired 1,5-disubstitued triazolylphosphine ligands **68**. The procedure could be performed in one pot by directly quenching the metalated triazole **66** with chlorophosphine. However, a separation of the triazole before phosphorylation makes purification of the final ligand easier [74].

**Scheme 13:**
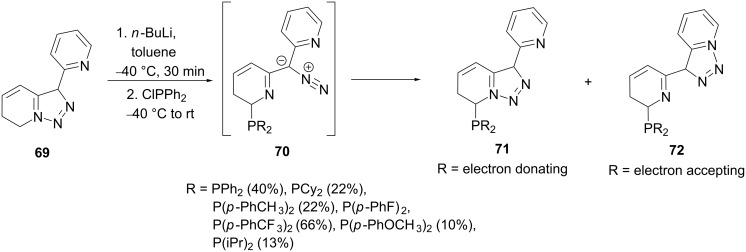
Synthesis of phosphanyltriazolopyridines and product selectivity depending on the substituents’ effects.

The direct *ortho*-metalation of pyridyltriazole **69** and subsequent reaction with chlorophosphines gave the isomeric ligands **71** and **72** in different ratios governed by the phosphine substituents (Scheme 13) [75]. When the R-substituent is more electron donating, the pyridine nitrogen *ortho* to the phosphine becomes more nucleophilic and intermediate **70** undergoes ring closure to give compound **71** with the phosphanyl substitutent in the 7-position of the fused ring structure. On the contrary, when the substituent R is electron withdrawing the pyridine nitrogen furthest away from the phosphine is more nucleophilic and hence is attacked resulting in isomeric ligand **72**. Thus triazolopyridines and quinolones can undergo ring-chain isomerism, which is dependent on the inductive and/or steric effects of the substituents present on the backbone [75–76].

**Scheme 14:**
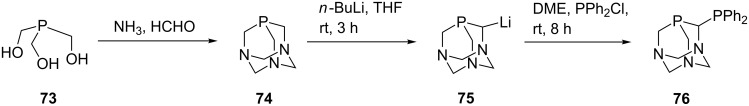
Synthesis of PTA-phosphine ligands.

The α-phosphorus methylene lithiation presents more prospects for the development of modified 1,3,5-triaaza-7-phosphaadamantane (PTA) ligands [77–78]. A chiral center is also introduced adjacent to the coordinating phosphorous [79]. The PTA-PPh_2_ ligand **76** is derived from the lithiated intermediate **75** (Scheme 14) [79]. The conversion of PTA (**74**) to the organolithium intermediate (PTA-Li, **75**) is almost quantitative with a 90% isolated yield. However, the yield of the desired ligand PTA-PPh_2_ **76** was very low after trapping **75** with diphenylchlorophosphine [79]. The PTA motif is water soluble, thermally, air and moisture stable. The PTA building block can be synthesized by coupling tris(hydroxymethyl)phosphine (**73**) with formaldehyde and ammonia in ice water or with hexamethylenetetramine.

**Scheme 15:**
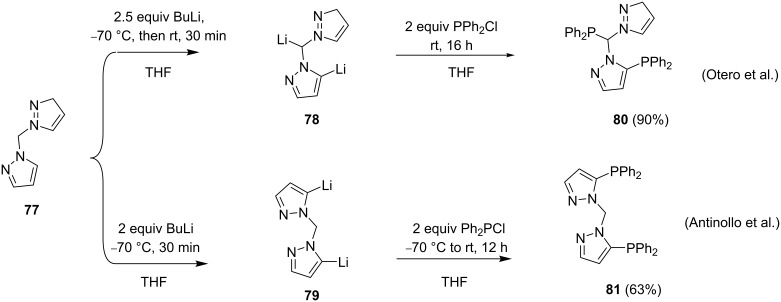
Synthesis of isomeric phosphine dipyrazole ligands by varying the reaction temperature.

A selective metalation can be achieved by varying reaction conditions and reagents. α-Lithiation of the methylene bridge and pyrazole ring in compound **77** allows for the synthesis of tris- and bis(pyrazole)phosphines. Antiñollo et al. [80] and Otero et al. [81] (Scheme 15) demonstrated the effect of varying the temperature on the synthesis of ligands **80** and **81**. Otero et al. [81] selectively obtained compound **80** when allowing the reaction mixture comprising compound **77** and 2.5 equiv *n*-BuLi in THF to warm to room temperature prior to reaction with chlorodiphenylphosphine at rt. On the other hand, when both steps, the lithiation and the introduction of the phosphine were performed at low temperature (−70 °C), compound **81** was obtained in 63% yield [80]. In both instances, the other isomer was present in minute quantities and could be separated by recrystallization.

Hybrid phosphine N-heterocyclic carbenes (NHCs) have proved to be versatile ligands in organometallic chemistry [82]. The synthesis of sterically crowded biaryl ligands is still a challenging task, especially under mild reaction conditions. The diphosphine complexes of imidazolylphosphines proved to be an alternative towards the coupling of sterically crowded biaryl ligands as they showed outstanding performances [8]. Phosphines with imidazole and imidazoline functional groups present some interesting features. The imidazolium functionality mimics active sites in biological molecules [83–84]. The ionic nature adds another dimension to the applicability of the catalysts in two-phase homogeneous catalysis because it allows easy recycling [85] and separation from the reaction mixture [8].

**Scheme 16:**
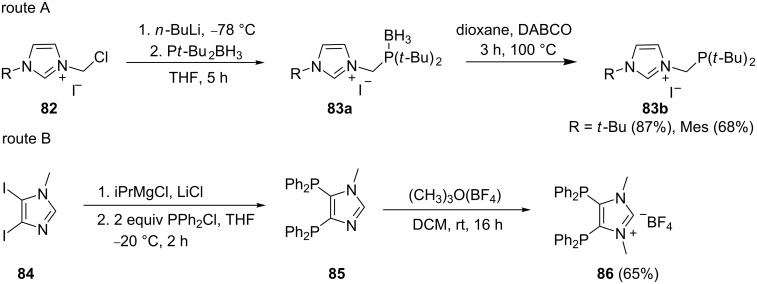
Synthesis of N-tethered phosphine imidazolium ligands (route A) and diphosphine imidazolium ligands (route B).

Carbon–halogen bonds are more activated than carbon–hydrogen bonds and hence the halogen is more labile and preferentially displaced. Brill et al. [86] took advantage of this fact by synthesizing a class of N-tethered phosphine imidazole ligands (Scheme 16, route A). The lithiation of the presynthesized chloromethylimidazolium iodide **82** and subsequent trapping with borane-protected di-*tert*-butylphosphine gave the imidazolium borane adduct **83a**. The subsequent deprotection then furnished **83b** in reasonable yields between 68 and 87%. Bis(diphenylphosphine)-substituted imidazoles were also synthesized by Karthik et al. [87] starting from the diiodoimidazole derivative **84**. The lithium chloride mediated magnesium/iodine exchange reaction of **84** followed by the addition of chlorodiphenylphosphine, afforded 1-methyl-4,5-bis(diphenylphosphino)imidazole (**85**). Finally, N-methylation gave the imidazolium salt derivative **86** in good yield (65%).

### Preparation of N-heterocyclic phosphines via metal-catalyzed P–C/N bond formation

**Scheme 17:**
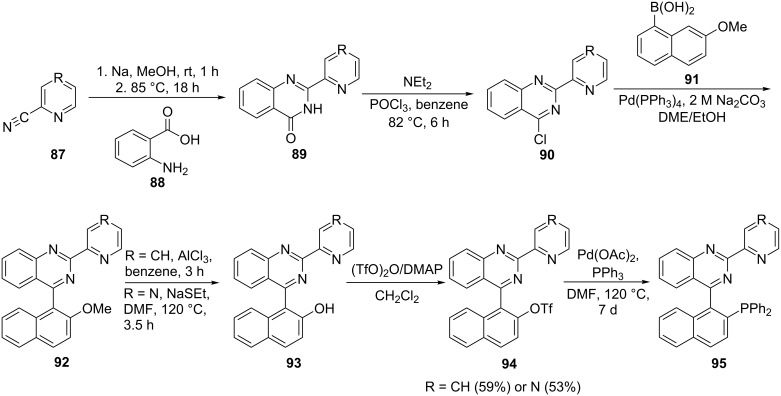
Synthesis of {1-[2-(pyridin-2-yl)- (R = CH) and {1-[2-(pyrazin-2-yl)quinazolin-4-yl]naphthalen-2-yl} (R = N) diphenylphosphine ligands **95**.

There is limited availability of certain N-containing precursors and hence they need to be synthesized through coupling of suitable pre-synthesized fragments. This, however, increases the number of synthetic steps making the procedure time consuming and unattractive for commercial use. The successful synthesis of configurationally stable diphenyl[(R-quinazolin‐4‐yl)(2-naphthyl)phosphines [R = 2-(2-pyridyl) and 2-(2-pyrazinyl] (QUINAP) by Brown et al. [88] as ligands for asymmetric catalysis instigated the synthesis of structurally similar ligands. Flanagan et al. [89] modified the QUINAP ligand by attaching 2-(2-pyridyl) (R = CH) and 2-(2-pyrazinyl) (R = N) moieties on the quinazoline ring (Scheme 17) and the phosphine was introduced via metal-catalyzed phosphorylation. The ligands were synthesized in eight steps with relatively good yield. The reaction between substituted nitrile derivatives **87** and anthranilic acid (**88**) catalyzed by sodium methoxide formed quinazolinones **89**. Subsequent chlorination of the quinazolinone resulted in the formation of 4-chloroquinazoline intermediates **90**. The subsequent Pd-catalyzed coupling of **90** and arylboronic acid **91** gave the methoxy intermediates **92** in reasonable yields. The demethylation of the 2-(2-pyridyl)methoxy intermediate was effected with aluminum chloride [90] and in case of the pyrazinyl derivative, sodium ethanethiolate [91] was used. The generated compounds **93** were then converted into triflate derivatives **94** by treatment with triflic anhydride in the presence of *N,N*-dimethyl-4-aminopyridine (DMAP) as the catalyst. Finally, the desired ligands were obtained by palladium-catalyzed phosphorylation with triphenylphosphine in DMF [92]. Resolution with palladium amine complexes and subsequent crystallization resulted in the enantiomerically pure ligands **95**.

*C*_2_-Symmetric atropisomeric diphosphines are among a diverse family of privileged chiral ligands in asymmetric catalysis [12]. In these compounds, the *C*_2_ axis of symmetry helps in increasing the selectivity of the formation of certain enantiomers by inhibiting other possible reaction pathways [93]. In particular, biarylphosphines and bidentate 2,2'-bis(diphenylphosphino)-1,1'-binaphthyl (BINAP) with a greater π-density and sterically demanding groups, have been extensively used in catalytic reactions [94].

**Scheme 18:**
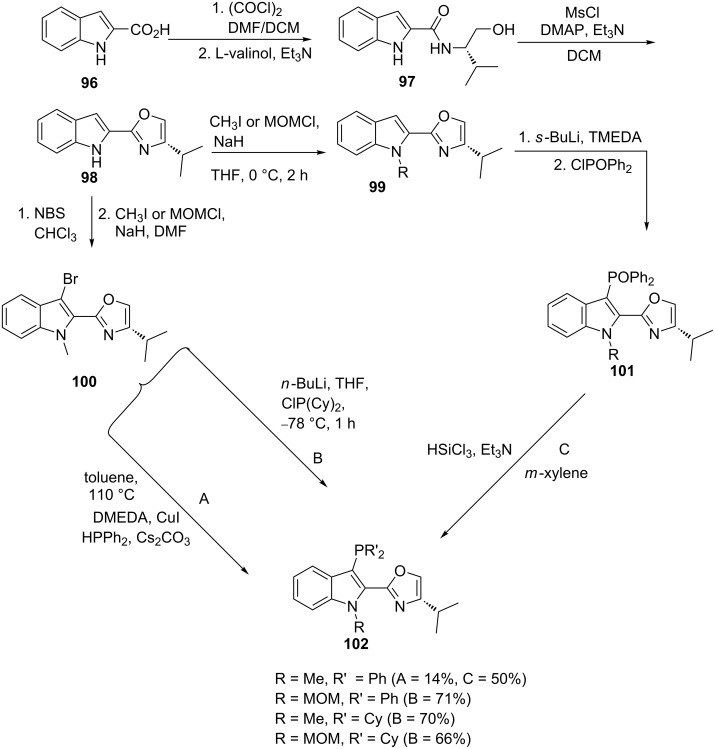
Synthesis of oxazolylindolylphosphine ligands **102**.

Wang et al. [95] reported a copper-catalyzed phosphorylation in the synthesis of an oxazolylindolylphosphine as shown in Scheme 18. The intermediate amide **97** was obtained by the reaction of ʟ-valinol with in situ-generated indolylacyl chloride. The latter compound was obtained through an oxalic acid-mediated chlorination of carboxylic acid **96** with dimethylformamide as catalyst in dichloromethane. Next, oxazoline derivative **98** was obtained via a methanesulfonyl chloride mediated cyclization of amide **97** and reaction with methyl iodide or methoxymethyl chloride afforded the N-substituted indole derivatives **99** (R = Me, MOM). The desired phosphinylated compounds **102** were obtained via three routes: The dicyclohexylphosphine derivatives **102a** and **b** were only accessible using the transmetalation route B and C from compound **100** and **101**, respectively. The phenyl phosphine derivatives, using the metal-catalyzed phosphorylation route A, and lower yields (14%) were obtained compared to the transmetalation route C.

#### P–H Bond addition to unsaturated precursors

**Scheme 19:**
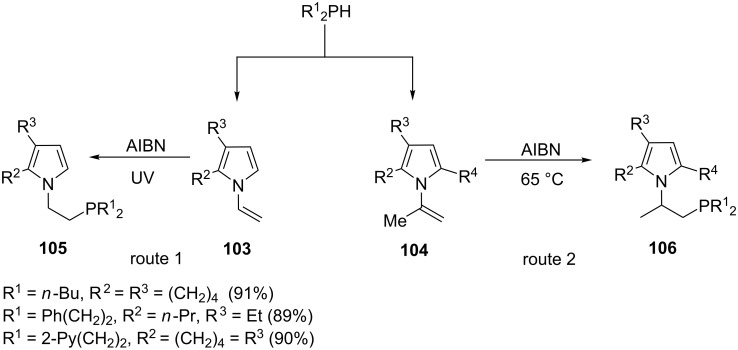
Synthesis of pyrrolylphosphine ligands.

The addition of P–H to unsaturated organic compounds (hydrophosphination) presents an atom economical, efficient and green strategy for the preparation of phosphines. The process can be initiated thermally, chemically or by UV irradiation. Radicals can also be used in hydrophosphination reactions. For example, azobisisobutyronitrile (AIBN) can initiate the addition of secondary phosphines to *N*-vinylpyrroles under heating or UV irradiation resulting in regio- and chemospecific adducts. Using the same approach Trofimov et al. [96] reported on the selective synthesis of tertiary diorganyl pyrrolylphosphines **105** and **106** in high yields starting from the corresponding *N*-vinylpyrroles **103** and **104** (Scheme 19). The *N*-isopropenylpyrrole precursor **104** gave the adducts with 100% regioselectivity. More recently a solvent and catalyst-free method has been reported for vinylpyridines [97].

#### Preparation of N-heterocyclic phosphines via P–N-bond formation

A P–N bond formation reaction is easier to be done than a P–C bond formation because the construction of the latter involves reaction conditions that are not suitable for multifunctionalized precursors. On the other hand, the installation of P–N bonds is usually done via a “one-pot synthesis” protocol. The quaternary salt byproduct that is formed when using an amine as the base can be easily separated by filtration. Bis(phosphine)amines with a P–N–P framework are more flexible to manipulate than diphosphines with a P–C–P framework [98]. The P–N–P cone angle and geometry on the phosphorus can be adjusted by changing the bulkiness of substituents around both, the N and P centers [99]. When reacting anilines and chlorophosphines under basic conditions they undergo P–N bond formation affording conventional aminophosphines [100–101]. A facile alternative method replaces the aniline with aminosilanes which produces trimethylchlorosilane as a byproduct which can be distilled off easily [102].

**Scheme 20:**
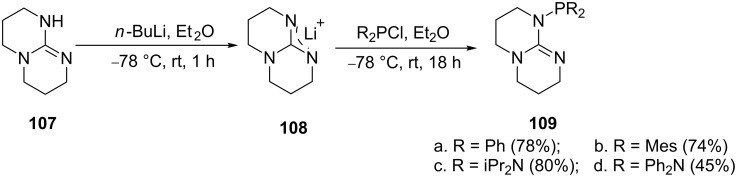
Synthesis of phosphine guanidinium ligands.

Bicyclic guanidine frameworks present an opportunity to form inflexible ligands that are inclined to exhibit a κ^2^-P,N-bonding mode in metal complexes. Dyer et al. [103] prepared cycloguanidine phosphine ligands (Scheme 20) using a one-pot procedure. First, the triazabicyclodecene **107** was metalated with *n*-butyllithium to give the intermediate **108** which was quenched with a chlorophosphine to produce the desired ligands **109** in excellent yields.

**Scheme 21:**
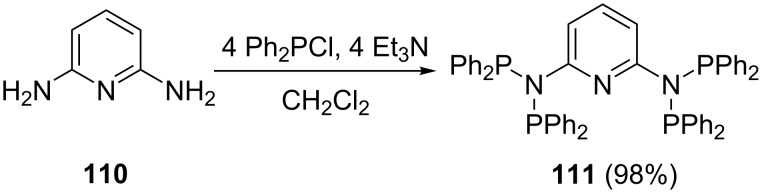
Synthesis of a polydentate aminophosphine ligand.

Besides substituents effects it has been reported that solvents may substantially influence reaction kinetics and product formation [102]. Biricik et al. [98] reported the preparation of polydentate aminophosphine **111** through a condensation–elimination–aminolysis reaction (Scheme 21). Reactions performed in diethyl ether and toluene resulted in bisphosphine imines and the reaction rates were low for anilines and analogous compounds. However, using dichloromethane proved to be a more suitable solvent because of higher product solubility and the reactions could be followed using ^31^P NMR spectroscopy [98,102]. The addition of four molar equivalents of Ph_2_PCl to a dichloromethane solution of 2,6-aminopyridine (**110**) afforded the multidentate ligand **111** in an excellent yield (97%) within 2 h. This in contrast to a method reported by Gaw et al. where the reaction took several days in diethyl ether [104].

**Scheme 22:**
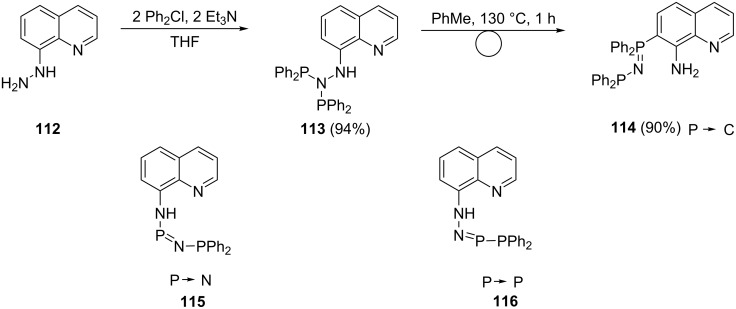
Synthesis of quinolylphosphine ligands.

Phosphine hydrazine P–N and N–N bonds are labile and can easily reorganize in the presence of some transition elements [105]. This provides an easy method towards the preparation of phosphazenides and phosphineamides [106]. In this way, Kornev et al. [106] prepared ligands **113** and **114** as shown in Scheme 22. The addition of chlorophosphine to 8-quinolyhydrazine (**112)** at a molar ratio of 2:1 in the presence of a base gave compound **113** in very good yield. Heating of compound **113** in toluene induced isomerization where the pendant arm is shifted to the quinoline ring via a P–C migration to obtain compound **114**. The corresponding P–N and P–P rearrangements were induced by reacting with zinc iodide and they could exist in the complexed state as structures **115** and **116**.

**Scheme 23:**
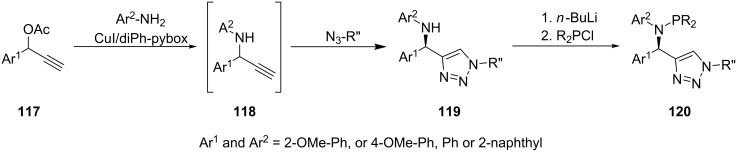
Synthesis of *N*-(triazolylmethyl)phosphanamine ligands.

**Figure 2:**
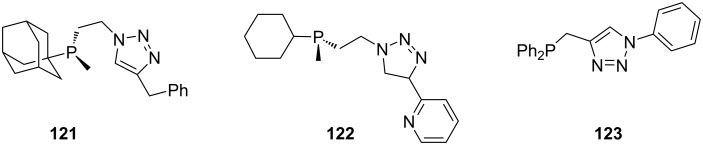
Triazolylphosphanamine ligands synthesized by Wassenaar’s method [22].

The substituents on the amine nitrogen affect the reaction conditions as well as the stability of the P–N bond. Wassenaar et al. [22] reported on a flexible click-phosphine ligand (**120**, Scheme 23) which could only be obtained by using a strong base such as *n*-BuLi for proton abstraction, probably due to a reduced acidity induced by the positive inductive effect of the substituents. The use of a weaker base such as triethylamine did not result in the targeted compounds and the authors attributed this to a stabilization of the NH proton by hydrogen bonds to the triazole nitrogen and methoxy oxygen atoms. The initial step in the synthesis of **120** is the enantioselective synthesis of the propargylamine **118** through the reaction of propargyl acetate **117** with the corresponding amine. This reaction is catalyzed by a copper(I) complex of 2,6-bis(4*R*,5*S*)-4,5-diphenyl-4,5-dihydrooxazol-2-yl)pyridine. The triazole amine **119** is obtained in situ by the reaction with the corresponding azide, which is catalyzed by the catalyst from the prior step. Finally, lithiation of compound **119** and addition of the corresponding chlorophosphines gave the phosphine triazole ligands **120**. Some ligands synthesized by the same route are included in Figure 2 (**121**–**123**).

P-stereogenic phosphine ligands are difficult to synthesize because of low configurational stability and less availability of P-stereogenic precursors. However, asymmetric synthesis can be used as strategy to introduce stereogenic P-atoms into the ligand’s backbone. The borane complexation approach is a unique stereoselective way for introducing a P-stereogenic center.

**Scheme 24:**
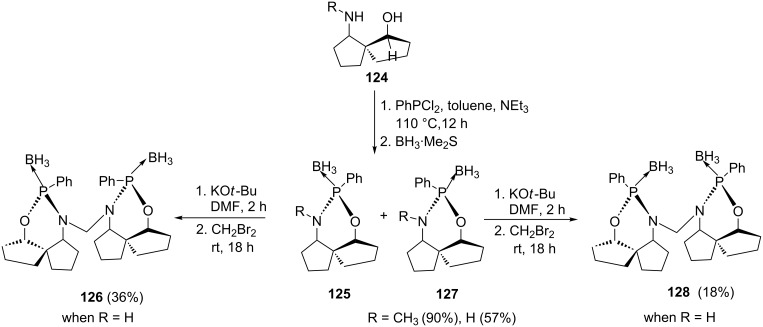
Synthesis of oxazaphosphorines.

Benoit et al. [2] reported on the synthesis of 2-phenyl-1,3,2-oxazaphosphorine ligands with a P-center and backbone chirality (Scheme 24). Spiro-1,3-amino alcohol compounds **124** were synthesized according to a literature procedure [107]. For the synthesis of the mono-*N*-methylated amino alcohol ligands a cooled solution of dichlorophenylphosphine was treated with triethylamine and mono-*N*-methylated spiro 1,3-amino alcohols **124**. The mixture was equilibrated under reflux allowing P-center inversion and an uneven mixture of diastereoisomers **125** and **127** was obtained. Treating the mixture with borane·dimethyl sulfide gave a mixture of diastereoisomers in a ratio of 2:5. The major isomer (+)-**125** was crystallized from (−)-**127** using an isopropanol/hexane mixture and confirmed to have *R*-configuration. The free ligand was obtained by deprotection of the P-center with 1,4-diazabicyclo[2.2.2]octane (DABCO) under refluxing in chloroform for two days at 50 °C.

Duplicating the same protocol with free amine spiro-amino alcohol derivative **124** gave compounds **125** and **127** (R = H) in low yields. An optimized procedure was used where dichlorophenylphosphine and borane·dimethyl sulfide in tetrahydrofuran were premixed at −78 °C. The temperature was then raised to 25 °C before neutralizing with triethylamine. Finally, spiro-1,3-amino alcohol was added and an equimolar mixture of compounds **125** and **127** was obtained with good yields. The dimeric ligands **126** and **128** were obtained by coupling each mono ligand in THF by first treating with potassium butoxide with subsequent addition of dibromomethane [2].

#### Substrate postfunctionalization and heterocycle construction

Some heterocyclic precursors can be readily obtained via accessible synthetic protocols. The nitrogen-containing compounds can be constructed and grafted on the phosphine precursor. Some available phosphines and organic precursors contain functional groups which can also be modified.

**Scheme 25:**
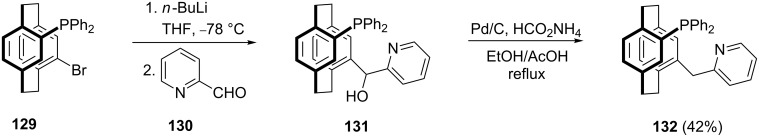
Synthesis of paracyclophane pyridylphosphine ligands.

Jiang et al*.* [108] attached a pyridyl moiety to a [2.2]paracyclophane phosphine support via a nucleophilic substitution reaction (Scheme 25). The nucleophile was generated by the addition of *n*-BuLi to enantiomerically pure [2.2]paracyclophane **129**. Subsequent addition of 2-pyridinecarboxaldehyde (**130**) afforded the hydroxy intermediate **131** with a high diastereoselectivity bias towards the (*R*_p_,*R*)-isomer. The racemic mixture could be separated by chromatography. Dehydroxylation of intermediate (*R*_p_,*R*)-**131** by using palladium on carbon as catalyst furnished planar chiral P,N-paracyclophane phosphine ligand **132** with a relatively low yield (42%). The [2.2]paracyclophane has proved to be an important support for planar chiral phosphine ligands. The ligands are generally rigid crystalline compounds that are stable in both high and low pH media and thermally stable up to 200 °C [109–110].

**Scheme 26:**
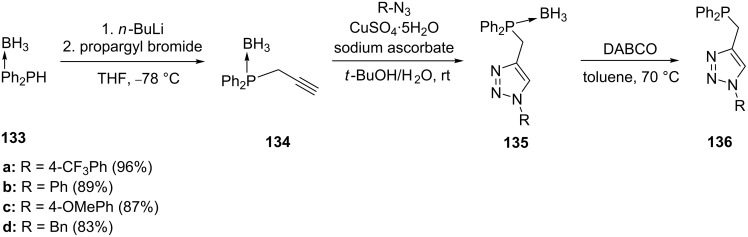
Synthesis of triazolylphosphine ligands.

**Figure 3:**
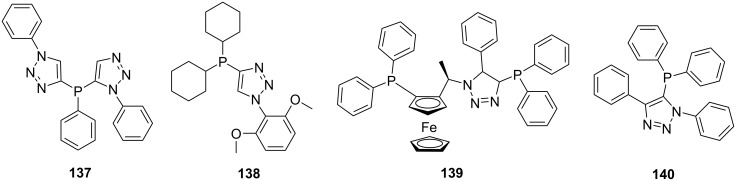
Click-phosphine ligands.

In these reactions the phosphine precursor can also be functionalized with appropriate groups for postfunctionalization. Detz et al. attached an alkyne to a phosphine which could easily be transformed to triazoles using click chemistry (Scheme 26) [111]. The click-phosphine ligands of type **136** were prepared by reacting phosphoacetylene **134** with different alkyl azides to generate the borane-protected ligand **135**. The protection is necessary because it prevents the formation of iminophosphorane during the click reaction. The click-phosphine ligands **136** can be liberated in excellent yields by reacting the protected ligands **135** with DABCO [111–112]. A diverse library of ligands prepared in a similar manner can be obtained by varying the phosphine and the substituents around the skeleton. Some of the ligands prepared include compounds **137–140** shown in Figure 3 [111–112].

**Scheme 27:**
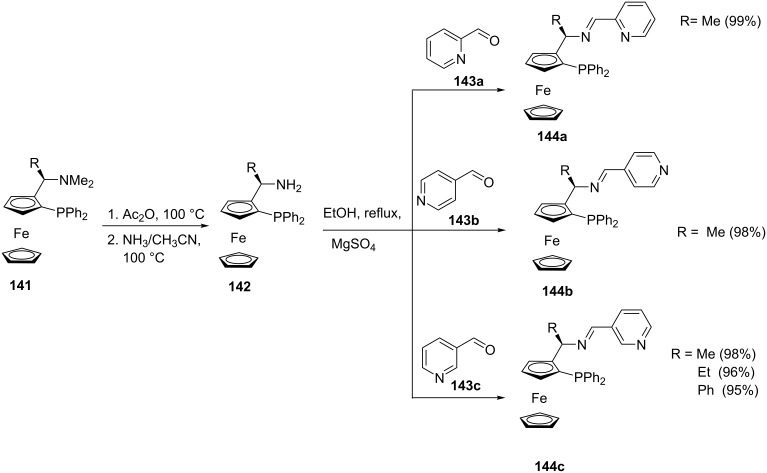
Ferrocenyl pyridylphosphine imine ligands.

Phosphines with amine functional groups can easily undergo Mannich condensation reactions. Ferrocene-based Schiff base ligands containing pyridine-*n*-yl ring (*n* = 2, 3, 4) (Scheme 27) were synthesized by Hu et al. [113] through the Mannich condensation of ferrocenylphosphine amine **142** and the appropriate pyridine carboxaldehyde **143** in refluxing ethanol/magnesium sulfate solution_._ The targeted ferrocenylphosphine imines **144** were obtained in almost quantitative yield. The α-ferrocenylethyl(dimethyl)amine **141** can be synthesized from ferrocenylethanol using phosgene and subsequent treatment with dimethylamine or, by using ferrocenyl(dimethylamino)acetonitrile. The phosphine group is introduced by *ortho*-lithiation of the ferrocenylamine followed by subsequent trapping with chlorophosphine [114–115].

**Scheme 28:**
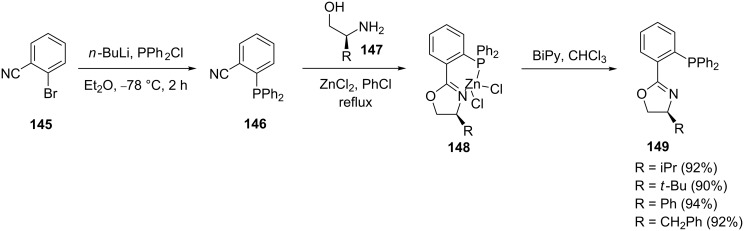
Synthesis of phosphinooxazolines (PHOX).

Preceding the ground breaking work by Pfaltz, many P,N ligands have been prepared for asymmetric catalysis [116]. However, the majority of them exhibited good to moderate enantioselectivity. The syntheses of chiral phosphinooxazolines was reported independently by Williams et al., Pfaltz and Helmchen, and Matt and Pfaltz [48,116–117]. Pfaltz et al. reported on the postfunctionalization in the synthesis of phosphinooxazoline (PHOX) ligands (Scheme 28) [48,117]. The 2-bromobenzonitrile (**145**) was treated with an in situ-generated phosphide reagent to obtain the 2-(diphenylphosphine)benzonitrile (**146**). This was then reacted with zinc chloride and aminoalkyl alcohol **147** in chlorobenzene to generate a Zn-oxazole complex **148**. Finally, ligand exchange with 2,2-bipyridine generated the desired 2-(diphenylphosphine)oxazole **149**.

Metallocenes have been used as ligand building blocks for many catalytic transformations. Especially, ferrocene has been used due to its high electron-donating capability and because it can be easily modified [118]. Furthermore, the ferrocenyl derivatives are reasonably stable and easily crystallize which makes purification much easier [119]. Ferrocene’s distinctive attributes, like explicit geometry and conformational adaptability, can orientate donor atoms prior to coordination making it ideal for syntheses of chiral ligands [118]. In the recent decades, Ugi’s amine has been one of the major interesting chiral ferrocenyl derivatives because the configuration at the α-ferrocenylmethyl position can be retained after nucleophilic substitution [115].

**Scheme 29:**
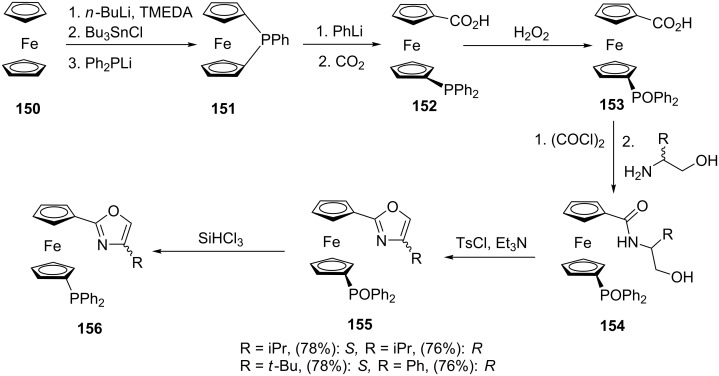
Synthesis of ferrocenylphosphine oxazoles.

Drahonovsky et al. [120] conveniently modified ferrocene to synthesize a series of ferrocenyloxazole ligands as depicted in Scheme 29. The ligand can be prepared from readily available ferrocene (**150**). The ferrocenophane **151** was prepared via a stannylferrocenyl derivative that was reacted with the phosphide. Subsequent reaction with carbon dioxide and phenyllithium gives the phosphine ferrocene carboxylic acid **152** as the major reagent. Oxidation of the phosphine using hydrogen peroxide generated the phosphine oxide **153**. In situ chlorination of the carboxylic acid followed by addition of the chiral amino alcohols gave the phosphoryl amido alcohols **154**. Cyclization in the presence of tosyl chloride/triethylamine yielded the analogous ferrocenyl phosphoryl oxazoles **155**, which were further reduced to give the corresponding phosphine oxazole ligands **156**. The ferrocenylphosphine oxazole ligand **156** is a fascinating example which contains three metal-centered chiral elements which are conferred upon coordination with a metal [121].

## Conclusion

In this review, the diversity of phosphine N-heterocyclic ligands and the variety of phosphine skeletons, which includes different five- and six-membered heterocycles and different coordinating sites has been reviewed. Different synthetic methods have been included which vary for different ligand systems. Some of the procedures satisfy more or less the following benchmarks, i.e., higher isolated yields and optical purity, allow variable substitution around the skeleton to adjust electronic and steric properties, use of low-priced and easily available reagents, mild and expedient reaction conditions, and few reaction steps. The motifs can also be chiral, and this is helpful in stereoselective synthesis. The introduction of different moieties can bring about enhanced properties like fluorescence, which can present possibilities for other interesting applications not only limited to organometallic catalysis. The combination of different heterocycles to make hybrid ligands can stimulate studies on their applicability in medicinal and OLEDs among other applications. In short, this review article presents the syntheses and architectures of phosphine *N*-heterocyclic ligands. Despite their success and many reported P,N-phosphine ligands, there is a need to designed new compounds to increase their library and to investigate other applications. It can be foreseen, that more probing and research on better synthetic protocols, which are fast, easy and greener, are needed. This is prime in the advancement of more flexible organometallic catalyst with novel applications.
